# Spinal ependymoma in adults: from molecular advances to new treatment perspectives

**DOI:** 10.3389/fonc.2023.1301179

**Published:** 2023-11-24

**Authors:** Giulia Cerretti, Federico Pessina, Enrico Franceschi, Valeria Barresi, Alessandro Salvalaggio, Marta Padovan, Renzo Manara, Vincenzo Di Nunno, Beatrice Claudia Bono, Giovanni Librizzi, Mario Caccese, Marta Scorsetti, Marta Maccari, Giuseppe Minniti, Pierina Navarria, Giuseppe Lombardi

**Affiliations:** ^1^ Department of Oncology, Oncology 1, Veneto Institute of Oncology IOV-IRCCS, Padua, Italy; ^2^ Department of Neurosurgery, IRCCS Humanitas Research Hospital, Milan, Italy; ^3^ Department of Biomedical Sciences, Humanitas University, Milan, Italy; ^4^ Nervous System Medical Oncology Department, IRCCS Istituto delle Scienze Neurologiche di Bologna, Bologna, Italy; ^5^ Department of Diagnostics and Public Health, University of Verona, Verona, Italy; ^6^ Department of Neuroscience, University of Padova, Padova, Italy; ^7^ Padova Neuroscience Center (PNC), University of Padova, Padova, Italy; ^8^ Department of Neuroscience, Azienda Ospedale-Università di Padova, Padua, Italy; ^9^ Department of Medicine - DIMED, University of Padova, Padua, Italy; ^10^ Department of Radiotherapy and Radiosurgery, IRCCS Humanitas Research Hospital, Milan, Italy; ^11^ Department of Radiological Sciences, Oncology and Anatomical Pathology, Sapienza University, Rome, Italy; ^12^ IRCCS Neuromed, Pozzilli, Italy

**Keywords:** ependymoma, spinal ependymoma, radiotherapy, chemotherapy, temozolomide, lapatinib

## Abstract

Ependymomas are rare glial tumors with clinical and biological heterogeneity, categorized into supratentorial ependymoma, posterior fossa ependymoma, and spinal cord ependymoma, according to anatomical localization. Spinal ependymoma comprises four different types: spinal ependymoma, spinal ependymoma *MYCN*-amplified, myxopapillary ependymoma, and subependymoma. The clinical onset largely depends on the spinal location of the tumor. Both non-specific and specific sensory and/or motor symptoms can be present. Owing to diverse features and the low incidence of spinal ependymomas, most of the current clinical management is derived from small retrospective studies, particularly in adults. Treatment involves primarily surgical resection, aiming at maximal safe resection. The use of radiotherapy remains controversial and the optimal dose has not been established; it is usually considered after subtotal resection for WHO grade 2 ependymoma and for WHO grade 3 ependymoma regardless of the extent of resection. There are limited systemic treatments available, with limited durable results and modest improvement in progression-free survival. Thus, chemotherapy is usually reserved for recurrent cases where resection and/or radiation is not feasible. Recently, a combination of temozolomide and lapatinib has shown modest results with a median progression-free survival (PFS) of 7.8 months in recurrent spinal ependymomas. Other studies have explored the use of temozolomide, platinum compounds, etoposide, and bevacizumab, but standard treatment options have not yet been defined. New treatment options with targeted treatments and immunotherapy are being investigated. Neurological and supportive care are crucial, even in the early stages. Post-surgical rehabilitation can improve the consequences of surgery and maintain a good quality of life, especially in young patients with long life expectancy. Here, we focus on the diagnosis and treatment recommendations for adults with spinal ependymoma, and discuss recent molecular advances and new treatment perspectives.

## 1 Introduction

Ependymomas are glial tumors that originate from the ependymal cells within the cerebral ventricles or in the spinal canal.

Primary spinal intradural tumors in adults are extremely rare entities and intramedullary neoplasms are even more rare, representing 5%–10% of all spinal tumors; spinal ependymomas are the most common adult intramedullary tumor, accounting for more than 60% of all intramedullary lesions (1).

The reported incidence of IDSCTs (intradural spinal cord tumors) is approximately 0.3 per 100,000 per year (2).

Men are slightly more affected than women (1.3:1), and there is a higher incidence in White individuals compared to Black individuals (3).

The location of the tumor largely depends on the patient’s age. While 90% of ependymomas are intracranial in children, 50%–60% of adult ependymomas occur in the spine (4). The cervical and lumbar portions of the spinal cord, including the filum terminale, are the most common sites of spinal ependymomas in adults (1).

Currently, no known environmental causes or specific oncogenic drivers have been identified. An increased incidence of ependymomas has been associated with the familial syndrome neurofibromatosis type 2. However, a Danish retrospective study reported that mutations in *NF1* and *NF2* were associated with ependymoma development in patients under 18 years old, but germline mutations were observed in fewer than 4% of cases (5).

Ependymoma survival rates are more favorable in adults than in children. The most recent population-based epidemiological data indicate a 10-year relative survival rate of 86.7% for adult ependymoma, with patients diagnosed under the age of 14 having a 10-year relative survival rate of 72% (6).

Because of the rare location, it is difficult to retrieve detailed information about epidemiology and survival outcomes in adult spinal ependymoma specifically (7).

Khalid et al. retrospectively assessed survival in 2,126 patients affected by spinal ependymoma; overall survival (OS) at 1 year, 3 years, and 5 years after diagnosis was 97.0%, 94.3%, and 93.3%, respectively (7).

Wostrack et al. demonstrated in a retrospective cohort of 158 patients with resected spinal ependymoma a 5-year progression-free survival (PFS) rate of 80% (1).

Mainly according to retrospective studies, risk factors for early progression of ependymomas include supratentorial location, histological grade 3, and subtotal resection (8). However, also site-specific molecular alterations may influence the prognosis. For this reason, the 2021 World Health Organization (WHO) classification of CNS tumors classifies ependymomas, not only on their histopathological features and anatomical site, but also on their molecular alterations (9). In addition, DNA methylation and gene expression profiling of ependymomas have identified at least nine subgroups characterized by distinct DNA methylation patterns and genetic alterations, which demonstrates the heterogeneity and complexity of these tumors (10, 11). Adult spinal ependymomas can be included in three out of nine of the methylation classes, specifically spinal subependymoma (SP-SE), spinal myxopapillary ependymoma (SP-MPE), and classic ependymoma (SP-EPN) (12).

## 2 Methods

An extensive thematic bibliographic research on the PubMed database was performed. English, pertinent articles, dating between 1984 and July 2023, have been identified.

The terms used for the PubMed search are: spinal ependymoma, spinal ependymoma in adult, methylation classes, target treatments in ependymoma, spinal symptoms, neurological outcomes, EANO guidelines, chemotherapy, radiotherapy, MYCN, temozolomide (TMZ), lapatinib, and WHO classification.

Only articles regarding the adult population and regarding ependymomas located in the spine have been included in this review.

## 3 Symptoms and clinical course

Given the low incidence of spinal ependymoma in adults, there are few reports on the symptoms and clinical presentation of the tumor (13–17) and studies aiming to distinguish the clinical features from other spine tumors or other diseases are missing.

Early symptoms are non-specific, and the onset is usually slow and progressive. The majority of patients had experienced symptoms for more than 6 months before the diagnosis, usually between 3 and 4 years (13–15, 18). A shorter duration of symptoms (from 2 weeks to 24 months) before diagnosis was observed for anaplastic ependymoma (19).

Sensory symptoms, in particular dysesthesias (numbness/tingling), are the most common (58%), followed by weakness (45%), back pain (35%), and radiating back pain (27%) (14–17). Sensory and motor impairments may determine gait abnormalities. Patients may also report aspecific symptoms as fatigue, drowsiness, or sleep disturbances (16).

Sensory complaints (numbness or tingling) are also the earliest to present in upwards of 70% of patients usually beginning distally at lower limbs with proximal progression. Pain at the level of tumor (rarely with a radicular distribution) may also occur early in the course of the disease progression (18). Sphincteric and sexual dysfunction also tend to occur early while weakness and associated spasticity usually occur late in disease progression and may be asymmetric. Motor involvement indicates that the tumor has significantly thinned the surrounding spinal cord (20). In anaplastic ependymoma, the most common symptom is pain, followed by sensory deficits, limb weakness, and sphincter dysfunction (19).

Tumor location affects the symptoms, with cervical ependymomas associated with upper limb involvement, thoracic ependymomas with symptoms at lower limbs, and lumbar ependymomas often presenting with back and leg pain, including radicular distribution, urogenital, and anorectal dysfunction. Lower limb impairment and sphincteric dysfunction may also be determined by cervical and thoracic ependymoma (18). Conus ependymoma is associated with pain, sexual dysfunction, and sphincteric disturbances as early symptoms, as well as progressive leg (one or two) weakness and/or numbness.

Cervical ependymomas present with sensory (more often) or motor deficit or isolated pain to one or both arms. Mild lower limb symptoms (weakness, spasticity, or sensory impairment) may be found at clinical examination at the moment of the diagnosis even if not reported by the patient. However, complaints at lower limbs may occasionally begin at the same time as upper limbs. Among presenting signs, atrophy of hands or proximal muscles may also be detected at clinical examination. Thoracic ependymomas present with weakness or numbness at lower limbs. Bladder or bowel disturbances may be reported at the diagnosis of both cervical and thoracic ependymomas.

Pain, sexual dysfunctions, and sphincteric disturbances as early symptoms are more common in conus ependymoma. Progressive leg (one or two) weakness and/or numbness are also associated with conus ependymoma. Notably, signs and symptoms at the moment of the diagnosis or in the course of follow-up may be asymmetric. Weakness may progress to para- or even tetraparesis with a subsequent loss of ambulation and a significant reduction of quality of life (17, 21) (**Table 1**).

**Table 1:** Signs and symptoms of spinal ependymoma.

Signs and symptoms	Location of spinal ependymoma		
	Cervical	Thoracic	Lumbar and conus
Dysesthesias (numbness/tingling)	+++ (Upper limbs)	++	++
Weakness	++ (Upper limbs)	+++	++
Spasticity	+	++	+
Back pain	+	+	++
Radiating back pain	+	+	+
Dysesthetic pain (“pins and needles” or burning pain)	+	+	+
Sphincteric and sexual dysfunction	++	++	+++
Fatigue	+	+	+

+/+++: from low to high frequency and impact.

The clinical course is usually slowly progressive with an accelerated progression in the months preceding the diagnosis. Acute worsening of symptoms is usually due to intratumoral hemorrhage, which represents a rare complication.

Neurological deficits are classified according to Modified McCormick grade (from I corresponding to minimal sensory symptoms to V corresponding to paraplegia or tetraplegia) and ASIA score (22). The majority of adult patients with spinal ependymoma have a pre-surgical modified McCormick grade I or II (15, 17, 23) or III in case of anaplastic ependymoma (19).

The most reliable predicting factor of post-surgical neurological outcome is the preoperative neurological state (13, 15, 24). Severe and long-term neurological deficits will not improve significantly after tumor resection, even if a gross total resection is reached (13). In the immediate post-surgery, patients may complain of a worsening of the previous symptoms of the onset of a new deficit, usually sensory, related to the posterior column traumatism caused by midline myelotomy, transient edema, or vascular compromise. In particular, patients may suffer from dysesthetic pain (“pins and needles” or burning pain) scarcely responsive to treatment, but self-limiting in few months (13, 17). After the transient worsening in the months post-surgery, usually patients improve at the level of their pre-operative deficits or may further improve, but some patients showed a worsening of their clinical status (19, 24). A progressive neurological worsening after surgery may underlie the presence of intramedullary cyst, tumor recurrence, or hematoma (15, 18).

A self-limiting orthostatic hypotension may occasionally occur after removal of upper thoracic and cervical ependymoma, thus interfering with the early mobilization, which is encouraged (13).

Because a risk of cerebrospinal fluid (CSF) dissemination exists for all patients with ependymoma, clinical and MRI evaluation should include both cranial and complete spinal districts. This extensive evaluation should be performed at the time of diagnosis with the aim of staging the disease and along the course of follow up. From a clinical point of view, it means that a comprehensive neurological evaluation should be performed including cranial nerves, cognitive functions, motor and sensory systems, gait, and cerebellar function; patients should be asked for new onset or worsening of headache, backpain, or radicular pain and sexual, bowel, bladder, and sphincteric disturbances (25–27). Late relapses may be asymptomatic; therefore, a meticulous neurological evaluation and enhanced MRI should be performed regularly in the follow-up (25, 27).

Considering the young age of these patients (usually in their 30s or 40s) and long life expectation, a satisfactory quality of life represents an important aim. In a study addressing this topic, almost half of the patients reported that the disease interfered at least moderately with work, general activities, walking, or enjoyment of life. Over 40% of patients stopped working or reduced their working hours due to the disease. Pain and weakness (and related accessibility constraints) represented the preeminent symptoms preventing the return to work. One-third of patients required help at home from a caregiver. Two-thirds of patients encountered difficulties when returning to sport activities due to pain, coordination problems, accessibility constraints, and fatigue symptoms (16, 17).

Low-grade ependymoma (specifically subependymoma and myxopapillary ependymoma) is often regarded as a tumor with a benign trajectory; however, many patients continue to have significant deficits and symptoms years after their initial diagnosis, and even in the best cases, ependymoma should be considered a chronic disease associated with symptom burden and high morbidity.

Among pharmacologic medications, patients are often treated for neuropathic pain with gabapentin and antidepressants (13); myorelaxants may be considered in case of spasticity. Early and intensive physical and occupational therapy optimize functional recovery and ultimately quality of life.

## 4 Histopathological and molecular features

In the last decade, molecular studies provide extensive evidence that different tumor types harbor distinct methylation profile, depending on their cell of origin and on the molecular alterations acquired during tumorigenesis (28). Therefore, DNA methylation profile, integrated with morphological and genetic features, was used to classify and diagnose tumors in the central nervous system (29).

In 2015, Pajtler et al., using DNA methylation profiling of 500 tumors, classified ependymal tumors in nine different molecular subgroups, across three anatomical compartments (supratentorial, posterior fossa, and spinal) (10). They showed that, in spite of similar histology, ependymomas arising in the spinal cord have a different DNA methylation profile, genetic alterations, and overall better clinical outcome, compared to those originating in the supratentorial compartment or in the posterior fossa (10). Ependymal tumors in the spinal cord were classified into spinal ependymoma, spinal subependymoma and myxopapillary ependymoma (10). However, a subsequent study demonstrated a distinct DNA methylation profile in a subgroup of spinal ependymomas featuring dismal prognosis in spite of aggressive treatment and displaying *MYCN* amplification (30). Therefore, the fifth edition of the WHO classification of CNS tumors categorizes spinal ependymal tumors into four different subtypes: spinal ependymoma, spinal ependymoma *MYCN*-amplified, myxopapillary ependymoma, and subependymoma (31).

**Figure 1:**
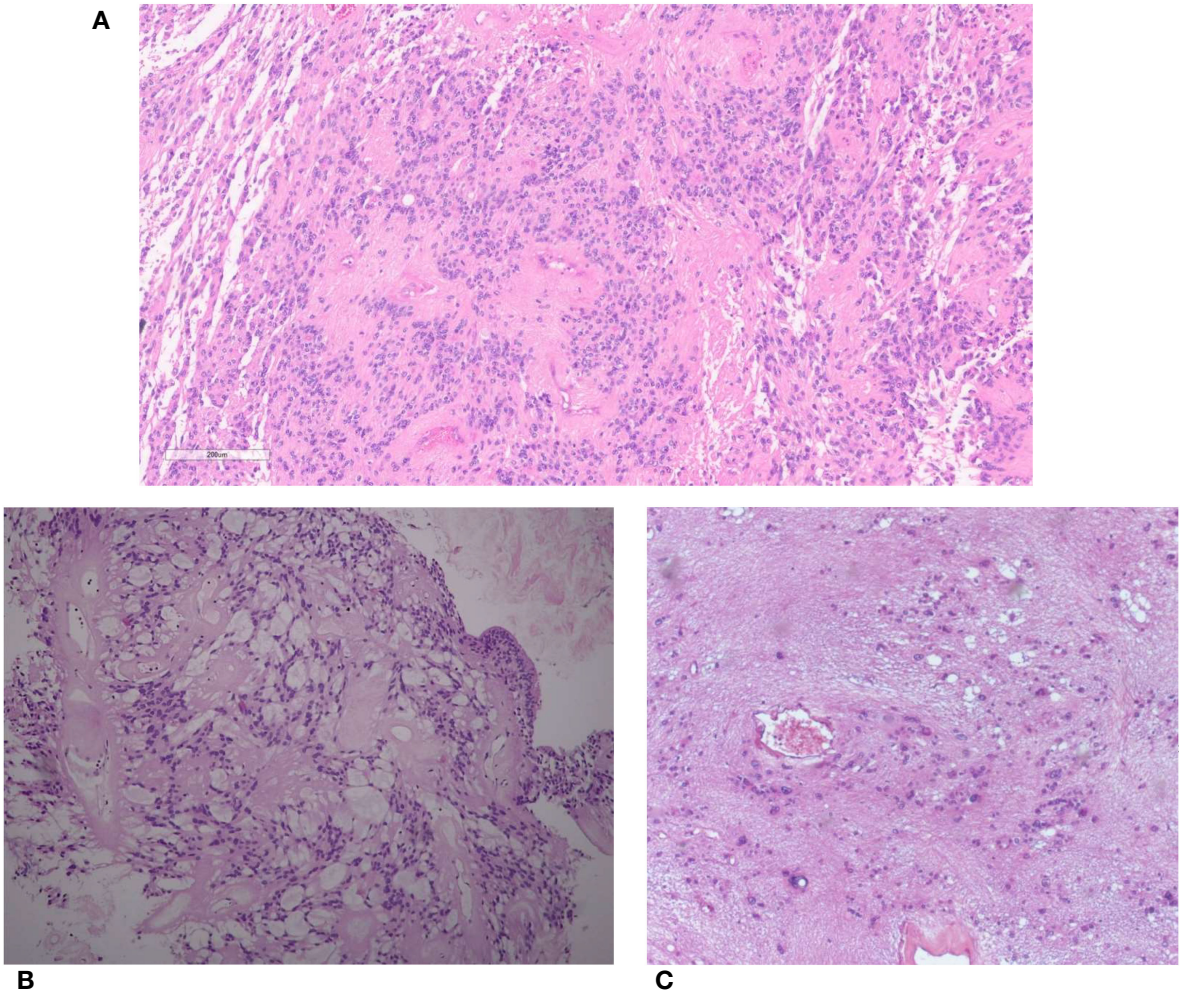
**(A)** Spinal ependymoma. Tumor cells with rounded to oval nuclei have fibrillary processes arranged around blood vessels (peri-vascular pseudorosettes). **(B)** Myxopapillary ependymoma. Tumor cells are arranged around blood vessels with interposed myxoid material. **(C)** Subependymoma. Tumor cells with uniform nuclei clusterized in a fibrillary matrix.

Spinal ependymoma is a well-demarcated tumor, histologically composed of monotonous glial cells, embedded in a glial fibrillary matrix, and showing rounded to oval nuclei. By definition, it lacks histopathological features of myxopapillary ependymoma or subependymoma and *MYCN* amplification (32). Tumor cells have fibrillary processes arranged around blood vessels and forming peri-vascular anucleate zones (so-called peri-vascular pseudorosettes) (**Figure 1A**). Only rare cases show true ependymal rosettes, consisting in glial cells arranged around empty lumina (32).

The papillary, clear cell, and tanycytic morphological variants, previously considered as subtypes of ependymoma, are now included as histopathological patterns (31).

Based on its morphological features, spinal ependymoma is graded into CNS WHO grade 2 and 3, the latter showing brisk mitotic activity and high cell density (32). The most frequent genetic alterations in this tumor subtype are chromosome 22q loss and *NF2* mutations (10) (**Table 2**).

**Table 2:** Spinal ependymal tumors.

Tumor Type	CNS WHO grade	22q loss, NF2 mutations
Spinal ependymoma	2 or 3, according to histopathological features	Main genetic alterations
MYCN-amplified spinal ependymoma	Not assigned, but aggressive clinical behavior	MYCN amplification MYC amplification*
Myxopapillary ependymoma	2	Genomic instability (grains)
Spinal subependymoma	1	19 loss, 6q loss

*Reported in one case (33).

CNS WHO grade and main genetic alterations.

Spinal ependymoma *MYCN*-amplified harbors a distinct DNA methylation profile and, by definition, *MYCN* amplification in tumor cells (34). It is a rare, recently defined tumor type with a higher incidence in women (1.7:1) and a median age at a diagnosis of 31 years (30, 35, 36), and a worse outcome compared with other spinal ependymomas. The reported median PFS after initial surgery and OS were 17 and 87 months, respectively, and it also undergoes frequent dissemination (30, 36).

Histologically, it features classical ependymoma morphology with peri-vascular pseudorosettes and true rosettes, but compared to spinal ependymoma, it invariably shows high mitotic activity, necrosis, and microvascular proliferation (34). In spite of its high-grade histological features and its adverse clinical outcome, it has yet to be assigned a definite CNS WHO grade. Spinal ependymoma *MYCN*-amplified is characterized by strong and intense MYCN immunostaining. Therefore, immunohistochemical staining for MYCN might be used as a screening method to identify this tumor subtype, although gene amplification should be confirmed using other methods, such as FISH analysis. Notably, a recent report described a case of a spinal ependymoma, with poor clinical outcome and DNA methylation profile consistent with ependymoma *MYCN*-amplified, lacking *MYCN* amplification and rather harboring *MYC* amplification (33). This suggests that the molecular portrait of spinal ependymoma *MYCN*-amplified may also include alterations in genes encoding for other proteins of the MYC family members beyond MYCN (**Table 2**).

*MYCN*, a member of the MYC group of oncogenes, codes for a transcription factor involved in the regulation of neuronal embryogenesis. It is involved in the oncogenesis of multiple tumor types, including neuroblastoma, pediatric glioblastoma, and medulloblastoma. The specific mechanisms by which *MYCN* promotes oncogenesis in spinal ependymomas have not yet been elucidated. Interestingly, multiple schwannomas have been reported in one patient with a *MYCN*-amplified spinal ependymoma, suggesting a possible relationship with neurofibromatosis type 2 (35, 36).

Myxopapillary ependymoma is a subtype almost exclusively occurring at the conus medullaris or filum terminale (37). It is defined by the presence of spindle or epithelioid neoplastic cells arranged around blood vessels (papillary feature) with interposed myxoid material (myxoid feature) or microcyst formation (38) (**Figure 1B**). This tumor was traditionally classified as WHO grade 1; however, reported rates of 10-year PFS of 60% and possible metastasization (39, 40) led to its reclassification as CNS WHO grade 2 in the fifth edition of WHO Classification (38) (**Table 2**).

Rarely, myxopapillary ependymomas can also develop outside of the CNS, usually within the sacrococcygeal or presacral tissues.

They are rare tumors with an incidence of 0.6–1.0 cases per 1 million person-years and a higher frequency in men (36, 41). Myxopapillary ependymomas are more common in adults with a bimodal peak approximately 25–29 years and 45–59 years. Overall prognosis is favorable (10-year survival rates > 90%) and complete resection is important for prognosis, but it can prove challenging (36, 41). CSF cytology is warranted before determining adjuvant treatments to exclude leptomeningeal dissemination (25). Although this is a rare event, distant metastases may also occur (36, 42). Spinal myxopapillary ependymoma is not characterized by specific genetic alterations, but it invariably features a high genomic instability, mainly consisting in copy number gains (10). Since this tumor displays HOXB13 upregulation, the immunohistochemical detection of this protein may be used in routine practice for the distinction from other tumor types (43, 44). A recent study on 185 tumors classified as myxopapillary ependymomas based on DNA methylation suggested that these diseases can be further subdivided into two methylation subtypes, named “A” and “B” (43). Tumors in subtype A were characterized by a higher number of copy number variations, papillary morphology, and worse outcome compared to tumors in subtype B (43). However, since methylation subtyping was not an independent prognostic factor after a multivariate survival analysis including histology, localization, and resection status (43), the utility of this distinction in clinical practice is to be determined.

Spinal subependymoma is a tumor preferentially localized at the cervicothoracic segment (45), and is histologically characterized by clusters of uniform to mildly pleomorphic tumor cell nuclei in an abundant fibrillary matrix (**Figure 1C**) and classified as CNS WHO grade 1 (46). It may show chromosome 19 and 6q loss, while other copy number alterations are infrequent (10, 12) (**Table 2**).

## 5 Neuroimaging in spinal ependymomas

Within the spinal cord, spinal ependymoma with and without *Myc* amplification, myxopapillary ependymoma, and subependymoma are four distinct WHO histologic subtypes. In spite of some imaging overlap, site, demographic, and distinct imaging features might provide useful clues about ependymoma subtype. Even though histologic evaluation remains necessary for differentiating these tumors from other tumor subtypes (e.g., astrocytoma, metastasis, lymphomas, etc.) and for detecting N-*Myc* amplification, neuroimaging (above all MRI) retains a pivotal role in defining tumor site, recurrence, or dissemination and in surgical planning.

### 5.1 Spinal ependymoma (with and without N-*Myc* amplification)

Spinal ependymomas can be found anywhere along the spinal cord but the distribution is not even: nearly half of ependymomas (44%) occur in the cervical cord, approximately 25% occur at the cervicothoracic junction, and 25% occur in the thoracic cord alone (47). As ependymomas arise from the central canal and are typically slowly expansive rather than infiltrative (48), they are characterized by a central spinal epicenter with a clear delimitation from the peripherally displaced normal cord. Vertebral canal widening, posterior vertebral body scalloping, and foraminal enlargement commonly confirm the slow tumor growth. Among neurofibromatosis type 2 patients, spinal ependymomas should be suspected as they represent up to 80% of intradural spinal tumor and affect 33%–53% of patients. Ependymomas usually extend less than astrocytomas (mean extension less than 4 vertebral segments).

Ependymomas are slightly and heterogeneously T2-hyperintense. Sagittal imaging best depicts the tumor features: markedly T2 hyperintense syringohydromyelia above or below the tumor is seen in 9%–50% of cases; peritumoral edema is present in approximately 2/3 of cases and tumoral cysts are present in approximately 1/5 of cases (49). Ependymoma have the tendency to bleed, leading to hemosiderin staining, i.e., a T2 hypointense rim at the caudal or cranial margins (“cap sign”), in 20%–33% of cases (48). This feature is suggestive of but not pathognomonic for ependymoma as it may also be seen in other tumors of the spinal cord (e.g., paragangliomas or hemangioblastomas). Superficial siderosis has also been reported (50, 51).

**Figure 2:**
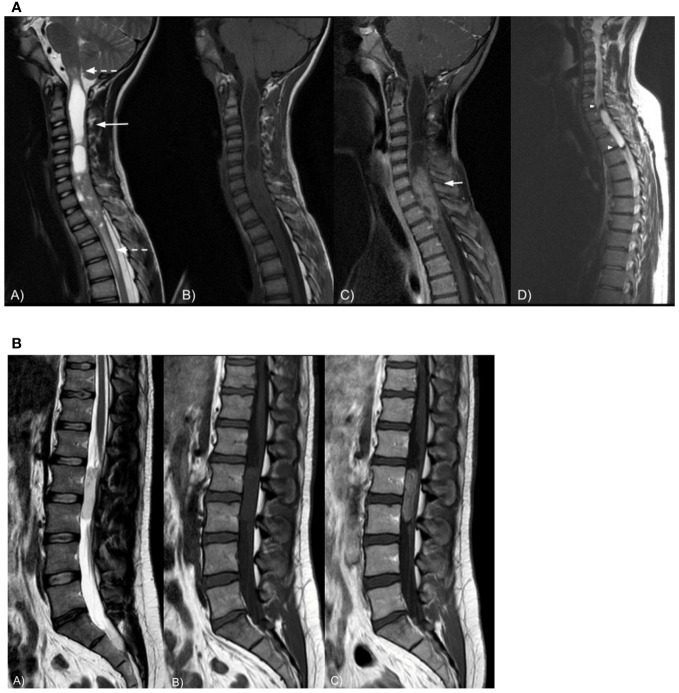
**(A)** Sagittal spine MRI in a 24-year-old female patient shows a T2-inhomogeneous (A), T1-isointense intramedullary (B) cervical mass with intratumoral cysts and syringomyelia (long arrow). The lesion is surrounded by moderate peritumoral edema (dashed arrows) and demonstrates strong contrast enhancement (C, short arrow). Sagittal T2-weighted spine MRI (D) in a 26-year-old male patient shows a cervico-thoracic intramedullary cystic lesion with marked peripheral hypointensity likely due to hemosiderin staining, i.e., the cap sign (arrowheads). Spinal cord ependymoma was proven at histopathology in both cases. **(B)** Sagittal spine MRI in a 34-year-old male patient shows a T2-hyperintense (A), T1-isointense (B) extramedullary intradural lumbar mass displacing the nerve roots of cauda equina posteriorly. The lesion demonstrates vivid and slightly inhomogeneous contrast enhancement (C). Myxopapillary ependymoma was proven at histopathology.

Most ependymomas are homogeneously T1-iso/hypointense; mixed-signal lesions reveal the occurrence of intratumoral cysts, necrosis, or hemorrhage. Ependymomas show vivid homogeneous enhancement. Calcifications are uncommon. In patients harboring N-*Myc* mutation, ependymomas show dismal prognosis due to a higher aggressivity and higher rate of tumor dissemination (**Figure 2A**).

### 5.2 Myxopapillary ependymoma

Myxopapillary ependymoma is extramedullary and intradural spinal tumor typically located in the lumbo-sacral region due to its origin in the filum terminale. Rarely, this subtype originates in the cervicothoracic region or fourth ventricle (52). Myxopapillary ependymoma can also extend into the neuroforamina; thus, differential diagnosis includes extradural tumors.

In the lumbo-sacral region, they typically appear as well-defined, encapsulated, oval, or sausage-shaped masses, often spanning more than one vertebra, though small oval tumors are also seen. The latter tend to displace the nerve roots of the cauda equina, while larger lesions often compress or encase them. Large, myxopapillary ependymomas may cause scalloping of the vertebral bodies and enlarge the spinal canal. Lesions are usually T1-isointense and T2-hyperintense. However, calcifications, hemorrhages, and cystic degeneration can be encountered (53), giving the mass an inhomogeneous aspect. Mucinous components occasionally result in T1 hyperintensity while calcifications might appear hyper- or hypointense. Hemorrhages often result in hypointense T2-hypointense tumor margins (cap sign) or, rarely, in brain, spinal cord, and nerve roots, surface T2-hypointense siderosis (54). Homogeneous contrast enhancement is common (**Figure 2B**).

### 5.3 Subependymoma

Spinal cord subependymoma is a rare, slow-growing, indolent, benign spinal cord tumor. It is radiologically similar to a spinal cord classic ependymoma as it is intramedullary, in the cervical or cervicothoracic region, typically T1 hypo- to isointense to white matter and T2 hyperintense. T2 signal might be heterogeneous and depends on the degree of cystic changes and associated hemorrhages or calcifications. However, subependymoma typically shows no or just slight enhancement. In addition, differently from ependymomas, the tumor growth is typically eccentric with the normal spinal cord included in the tumor (bamboo leaf sign in 76%) (55).

## 6 Surgery in spinal ependymoma

### 6.1 Indications for surgical treatment

According to the most recent European guidelines (3, 25), the gold standard of treatment for any suspected spinal ependymoma is surgical resection (25), with the aims of obtaining the tumor’s histomolecular characterization, achieving complete resection (3), whenever it is possible, and preserving/improving the patient’s functional status (56). Indeed, in both pediatric and adult spinal ependymomas, tumor grading and the extent of resection seem to be, to date, the only independent factors capable of carrying a significant impact on patient prognosis (3, 56–59).

### 6.2 Intraoperative setup and surgical technique

In spinal ependymomas, the extent of resection depends on several factors, including tumor location and volume, the presence of a capsule, and histomolecular grading (56–60).

Thanks to advances in modern microsurgical techniques, a gross total resection while maintaining/improving the patient’s functional outcome is possible in most cases (84%–93%) also because these tumors only rarely infiltrate the spinal cord (56, 57, 61).

#### 6.2.1 Intraoperative neurophysiologic monitoring

Intraoperative neuromonitoring, including motor evoked potentials (MEPs) and somatosensory evoked potentials (SEPs), is of utmost importance to prevent and reduce the risk of postoperative neurological deficit related to surgical maneuvers (62). The D-wave monitoring, whose electrodes can be placed either subdural or epidural, allows recording the corticospinal activity without any peripheral conductivity-related artifacts, and it is the most specific predictor of postoperative transient/permanent motor deficit (63).

#### 6.2.2 Soft tissue opening and laminectomy

A standard posterior midline approach is usually tailored according to the tumor extension. A posterior laminectomy is usually performed including one level above and one level below the tumoral mass, sparing the articular facets to avoid any long-term segmental instability. In this regard, intraoperative neuronavigation (intraoperative ultrasound, CT-MRI-based neuronavigation) can be employed during the initial phases of the procedure to precisely locate the tumor and tailor its exposure.

#### 6.2.3 Durotomy and myelotomy

Under microscopic view, a midline durotomy is performed and the dura is suspended laterally with the aid of suture stitches. The arachnoid is then sharply and widely opened to fully expose the posterior spinal cord surface. At this point, the tumor is usually visible and well-distinguishable from the normal tissues. Neuronavigation could be employed, again, in cases of non-superficial tumors.

#### 6.2.4 Resection technique

Whenever possible, *en bloc* resection must be preferred over piecemeal resection (which is usually performed with the aid of an ultrasonic aspirator) to minimize the risks of CSF dissemination (63). A midline posterior myelotomy is performed under neuromonitoring guidance. Tumor dissection from the normal tissue is then performed both sharply and bluntly with the aid of micro scissors, micro forceps, and micro dissectors. The dissection plane must always be preserved and carefully maintained.

Tumors with more distinct capsules are associated with fewer post-operative neurologic deficits, likely because they are more easily separated from normal tissue.

#### 6.2.5 Closure

Careful hemostasis is performed at the end of the resection with constant irrigation, which should be preferred over bipolar coagulation to avoid any inadvertent ischemic injury to the nearby eloquent structures. A watertight dural closure is performed to minimize the risk of postoperative CSF leakage. Autologous fat tissue and/or synthetic materials can also be used to strengthen the dural seal.

### 6.3 Surgical considerations

The association between extent of resection (EOR) and PFS in patients with spinal cord ependymoma has been diffusely recorded in retrospective studies (64, 65) (**Table 3**). However, evidence of definitive relationships with PFS or OS are lacking. In a large review, Oh et al. analyzed 175 patients with SCE across 43 different studies, focusing on the association between tumor grading and outcome. For the entire cohort, maximal resection was associated with better outcome even after controlling for adjuvant radiation therapy. In the group of patients with grade II ependymomas, those with gross total resection (GTR) had a significantly lower recurrence rate compared to patients with subtotal resection (STR), while patients with myxopapillary ependymoma showed a similar recurrence rate in case of GTR and STR (12.1% vs. 26.1%), respectively (60).

**Table 3:** Studies analyzing surgery in spinal ependymoma.

Study	N pts	Grade WHO N pts	Site	EOR	Postoperative neurological deficit	RT N pts	PFS	OS
Volpp PB 2007 (59)	23	IDSCT	Extramedullary 23	GTR 9 STR 14	–	6	5-year PFS Surgery alone 94% GTR+RT 100% STR+RT 100%	5-year OS 77%
Oh MC, 2013 (66)	348	Grade 2 337 Grade 3 11	–	GTR 268 STR 80	–	57	mPFS GTR nr STR 48 ms STR+RT 96 ms 5-year PFS GTR 97.9% STR 45.1% STR+RT 65.3% *p* < 0.001	5-year OS GTR 98.8% STR 73.7% STR+RT 79.3
Skrap B 2021 (62)	100	-	Intramedullary 100	GTR 89 STR 11	Stable/improved 84 Deterioration 16	0	–	–
Lee SH 2013 (64)	88	Grade 2 61 MPE 24 Grade 3 3	Intramedullary 59 Extramedullary 29	GTR 72 STR 15 PR 1	Stable/improved 52 Deterioration 36	20	10-year PFS GTR 95% GTR+RT 89% STR 43% STR+RT 22% *p* = 0.541	–

N, number; WHO, World Health Organization; EOR, extent of resection; RT, radiation therapy; PFS, progression-free survival; OS, overall survival; MPE, myxopapillary ependymoma; GTR, gross total resection; STR, subtotal resection; PR, partial resection; B, biopsy; Gy, gray; S, surgery; CSI, craniospinal irradiation; nr, not reached; ms, months. All of the studies were performed retrospectively.

For the grade III ependymoma group, GTR strongly impacted outcome (no recurrences among patients underwent maximal resection compared to 80% of patients with STR). The small population in the study represents a relevant limit for this results interpretation (60).

Lee et al. showed that GTR alone is a good treatment strategy for spinal ependymomas. Early diagnosis and surgery, before severe paralysis, are important to obtain good functional outcomes. Subtotal resection with radiation therapy for intramedullary lesions appears to offer no advantages over gross total removal (64).

### 6.4 Complications

Despite efforts to preserve normal tissue, post-operative neurologic deficits are unfortunately possible, with risk best predicted by the patient’s baseline neurologic function. Compared to patients with deficits at baseline, patients with good neurologic function before surgery showed a lower risk of post-operative neurologic impairment (67). Thoracic tumor location, related to the region’s relatively limited blood supply, are more likely to worsen post-operative status (57, 65). Interestingly, extent of surgical resection has not been found to be related to post-operative neurologic function (67).

N, number; WHO, World Health Organization; EOR, extent of resection; RT, radiation therapy; PFS, progression-free survival; OS, overall survival; MPE, myxopapillary ependymoma; GTR, gross total resection; STR, subtotal resection; PR, partial resection; B, biopsy; Gy, gray; S, surgery; CSI, craniospinal irradiation; nr, not reached; ms, months. All of the studies were performed retrospectively.

## 7 Radiation therapy in spinal ependymoma

The role of radiation therapy in spinal ependymoma is still a topic under investigation due to the lack of results provided by prospective or randomized trials (**Table 4**). The large part of published studies are retrospective, with a limited number of patients included, grade 2 and 3 together, in which a small percentage of patients received adjuvant RT. Notwithstanding, RT has proven to be an effective local treatment in spinal ependymoma; above all, in cases of incomplete surgical resection, available data related to the benefit on survival are discordant.

**Table 4:** Studies analyzing radiation therapy in spinal cord ependymoma.

Study	N pts	WHO grade N pts	Disease site	Surgery EOR	N pts treated with RT for EOR	Total doses (range) Site	PFS	OS
Wostrackt M. 2018 (1)	158	Grade 1 44 Grade 2 105 MPE 35 Grade 3 9	Focal 132 Multifocal 26	158 GTR 127 STR 21 PR 10	15 GTR 6 STR 5 PR 4	40–60 Gy	5-year PFS 80% mPFS GTR nr STR 60 ms PR 43 ms S+RT 43 ms *p* = 0.079	–
Wahab SH 2007 (68)	22	Grade 2 13 MPE 9	Focal 13 Multifocal 9	22 GTR 2 STR 20	22 GTR 2 STR 20	45 Gy (30–54 Gy) Local RT 13 CSI 3 Whole spine 6	5-, 10-, and 15-year PFS 80%	5-year OS 85% 10-year OS 78% 15-year OS 64%
Gomez DR 2005 (69)	37	Grade 1–2 33 Grade 3 4	Focal 30 Multifocal 7	37 GTR 4 STR 20 B 13	37 GTR 4 STR 20 B 13	50.4 Gy (45–54 Gy) Local RT 31 CSI 4 Whole spine 2	mTTP 82 ms 5-year PFS 75% 10-year PFS 50% 15-year PFS 46%	5-year OS 83% 10-year OS 64% 15-year OS 61%
Whitaker SJ 1991 (70)	58	Grade 1 40 Grade 2 7 Grade 3 6 No grade 5	Focal 34 Multifocal 24	58 GTR 14 STR/PR 33 B 11	43 GTR 2 B/PR/STR 41	50 Gy (30–55 Gy) Local RT 25 CSI 12 Whole spine 6	5-year PFS S+RT 9% 10-year PFS S+RT 54% 5- and 10-year PFS S alone 92% Not statistically significant	5-year OS S+RT 69% 10-year OS S+RT 62% 5,10-year OS S alone 92% Not statistically significant
Oh MC, 2013 (66)	348	Grade 2 337 Grade 3 11	–	348 GTR 268 STR 80	57 GTR 10 STR 47	>50 Gy or <50 Gy No differences on PFS or OS	mPFS GTR nr STR 48 ms STR+RT 96 ms 5-year PFS GTR 97.9% STR 45.1% STR+RT 65.3% *p* < 0.001	5-year OS GTR 98.8% STR 73.7% STR+RT 79.3
Byun HK 2018 (71)	25	Grade 1 12 Grade 2 12 Grade 3 1	Focal 19 Multifocal 6	25 STR 19 B 6	25 STR 19 B 6	50.4 Gy (44–59.4 Gy) Local RT 19 CSI 5 Whole spinal+PF 1	5-year PFS 70.8%	5-year OS 83.7%
Shaw EG 1986 (72)	22	Grade 1 19 Grade 2 2 Grade 3 1	Focal 14 Multifocal 8	22 GTR 8 STR 11 B 3	22 GTR 8 STR 11 B 3	50 Gy (36–57 Gy) Local RT 17 CSI 5	5-year DFS 81% 10-year DFS 71%	5-year OS 95% 10-year OS 95%
Lin YH 2005 (73)	20	Grade 1–2 13 MPE 6 Grade 3 1	Focal 20	20 GTR 14 STR 6	6 STR 6	50–60 Gy Local RT 6	LC 19/20 pts	mOS GTR 104 ms STR+RT 135 ms
Akyurek S 2006 (74)	35	MPE	Focal 30 Multifocal 5	35 GTR 21 STR 13 B 1 Unknown 1	22 GTR 10 STR 11 B 1	50.4 Gy (44.3–56 Gy) Local RT 17 CSI 5	5-year PFS S alone 49% S+RT 82% 10-year PFS S alone 37% S+RT 75% 10-year LC S alone 46% S+RT 86% GTR 58% GTR+RT 90% STR 0% STR+RT 90%	–
Weber DC 2015 (40)	183	MPE	Focal 179 Multifocal 4	182 GTR 99 STR 73 B 6 Unknown 5	86	50.4 Gy (25–60 Gy) Local RT 82 CSI 4	10-year LC Surgery alone 45% Surgery + low-dose RT 71% Surgery + high-dose RT 66% 10-year PFS Surgery alone 38% Surgery + low-dose RT 56% Surgery + high- dose RT 66%	10-year OS Surgery alone 92% Surgery + low-dose RT 86% Surgery + high-dose RT 71%

N, number; WHO, World Health Organization; EOR, extent of resection; RT, radiation therapy; PFS, progression-free survival; OS, overall survival; MPE, myxopapillary ependymoma; GTR, gross total resection; STR, subtotal resection; PR, partial resection; B, biopsy; LC, local control; Gy, gray; S, surgery; CSI, craniospinal irradiation; nr, not reached. All of the studies were performed retrospectively.

The results of the multicenter retrospective study led by Wostrack et al. that evaluated 158 patients with spinal ependymoma did not show an improve survival in patients receiving adjuvant RT following subtotal resection, although only 15 cases received RT (1). Data from the SEER database in which a deep learning algorithm was applied to >2,200 patients with spinal ependymoma, assessing predictive factors of OS, identified RT as an independent predictor (75). To date, suggestions regarding the use of adjuvant RT and total doses to deliver widely varied in relation to histological subtype, grade, and extent of surgical resection (66, 68–70, 76).

For *grade 1 spinal subependymoma*, RT is not indicated given the possibility of obtaining complete surgical resection in almost all patients, the favorable patients prognosis, and the low incidence of local recurrence.

In *grade 2 ependymoma* patients receiving gross total resection (GTR), adjuvant RT does not improve PFS and OS, and accordingly is not recommended. On the other hand, when GTR is not feasible because of infiltration of spinal cord or nerve roots, the recurrence rate reaches up to 50%–70% without adjuvant therapy, with a 5-year survival rate of 73.7%. A comprehensive review of the literature on 348 WHO grade 2 and 3 classic spinal cord ependymoma patients has been conducted aiming to evaluate whether adjuvant RT improves tumor control in patients who underwent surgical resection (66). Patients who received GTR were 77%, and 33% received STR; among these, adjuvant RT has been administered in 58.8% of the cases. On univariate and multivariate analysis, the use of adjuvant RT after STR significantly affected PFS [*p* < 0.001; hazard ratio (HR) =2.26, *p* = 0.047]. By contrast, improved OS was only associated with GTR (GTR versus STR + RT group; HR = 0.07, *p* = 0.001) and grade 2 ependymomas (HR = 0.16, *p* = 0.001). No correlation between RT doses employed (≤50 Gy vs. >50 Gy) on PFS or OS has been recorded. This high recurrence rate has promoted the use of adjuvant RT for patients receiving incomplete surgical resection (72).

The role of radiotherapy for *Myxopapillary ependymoma* (*MPE*) in adults, reclassified by the 2021 WHO classification of CNS tumors as *grade 2*, is controversial too. Unlike the other grade 2 ependymomas, MPE typically located in the conus medullaris, cauda equina, and filum terminal region has a tendency towards a leptomeningeal spread occurring in up to 10% of the initially localized disease. Owing to the close adhesion to the nerve roots and the production of a myxoid matrix, a safe GTR of MPE may be challenging with a GTR ranging from 53% to 75% (77, 78). In this context, the role of RT is crucial. Mixed results provided by retrospective series are available, some did not demonstrate a benefit in the recurrence rates adding adjuvant RT, and others underlined the superiority of a combined local treatment approach, consisting of surgery followed by RT over surgery resection alone (40, 73, 74, 79). The experience of the MD Anderson Cancer Center showed that the addition of postoperative radiotherapy to surgery was associated with significantly longer 10-year PFS rates (75% for the combination vs. 37% for surgery alone) (79). Among 11 patients who received GTR alone, 5 (45%) had recurrence. A total of 12 (34%) patients had disease recurrence, all in the neural axis; 8 of them had treatment failure at the primary site only, 3 in the distant neural axis only, and 1 at the primary site and in the distant neural axis. Patient age (>35 years; *p* = 0.002) and adjuvant RT (*p* = 0.04) significantly affected PFS. The larger published series evaluating the outcome of 183 MPE patients underlined the use of adjuvant RT as the factor that allowed an increase of the 10-year PFS from 40% to 70% in patients receiving a combined treatment with respect to those treated with surgery alone. This would justify a more liberal use of adjuvant RT, especially in those patients with subtotal resection and piecemeal resection, or questionable GTR in patients with spinal MPE (40). In summary, although adjuvant RT may not ultimately affect OS, decreasing recurrence can appreciably benefit patient outcomes by avoiding repeated surgeries, which are associated with significant morbidities.

Grade *3 spinal ependymomas* are rare entities, accounting for between 2% and 8% of all spinal ependymomas, and the patient prognosis is poor (71). Postoperative radiotherapy is indicated regardless of the extent of resection as suggested by international guidelines (25).

The optimal dose of radiation for spinal cord ependymomas also remains to be determined. Most authors currently recommend doses of 45–54 Gy with long-term follow-up because recurrence can occur many years after initial treatment. High doses (≥50 Gy) have been proven to increase the local control and PFS (10-year PFS from <40% to 70%) with good tolerance and without substantial late toxicity (40, 66, 79). In cases of cranial and spinal dissemination, craniospinal irradiation (CSI) of 36 Gy is recommended with a boost up to 45–54 Gy on local lesions (25). However, the recommended dose of radiation has not been clearly defined because high doses of radiation may be associated with increased risk of radiation myelopathy (40, 71, 79).

## 8 Systemic treatment for ependymomas of the spinal cord

Ependymal tumors of the spinal cord are more common in adults than in children and have a better prognosis as compared to spinal cord astrocytoma (25, 80, 81). Owing to the rarity of these tumors, there are very little data investigating the clinical impact of systemic therapy (82) (**Table 5**).

**Table 5:** Studies evaluating systemic therapy in patients with spinal cord ependymoma.

Study	Type	N. pts	Agent	Outcomes
Gilbert, 2021 (83)	Prospective	25 spinal (50 patients in total)	TMZ (dose dense) + lapatinib	12% complete/partial response 88% stable/no response/progression Median OS 40.7 months
Chamberlain, 2002 (84)	Prospective	10	Etoposide	20% partial response Median OS 17.5 months
Kim, 2011 (85)	Case report	2	RT + Temozolomide	Median OS 12–39+ months
Fujiwara, 2018 (86)	Case report	1	TMZ	Complete response Median OS 72+ months
Tapia Rico, 2020 (87)	Case report	1	Tiselizumab	Stable disease Median OS 28+

OS, overall survival.

To date, no prospective studies investigated the role of adjuvant systemic treatments. The only experience of concurrent radiation and TMZ chemotherapy is represented by a small case series. In one of these studies, a single patient with anaplastic spinal cord ependymoma had an OS of 39 months (88). A similar OS was observed in another distinct series (89). Thus, the only setting where systemic treatments have been investigated is the advanced disease refractory to loco-regional treatments. In 2020, Gilbert MR et al. published the results of a prospective phase II study investigating the combination between dose-dense TMZ and lapatinib within adult patients with recurrent ependymoma (83).

In this single-arm study, 50 patients received TMZ at a dose of 125 mg/m (2) as a single daily dose on days 1–7 and 15–21 of a 28-day cycle in combination with a single daily dose of lapatinib 1,250 mg orally.

Between the 50 patients enrolled, 25 (50%) had a spinal cord ependymoma with a variable tumor grade. In particular, 7 patients had anaplastic grade 3 tumors, while 16 patients had grade 2 (*n* = 8) and grade 1 (*n* = 8) spinal cord ependymomas; and 2 of unknown grade (83). The primary endpoint of the study was the median mPFS. In the overall cohort, after a follow-up of 4.41 years, the mPFS was 7.8 months (95% CI: 5.5-12.2) and median OS was 2.25 years (95% CI: 1.7–3.97 years). Tumor response was observed in eight patients (16%) consisting of two complete and six partial responses (83).

In patients with spinal cord tumors, the median PFS was 0.9 years (95% CI: 0.46–1.84 years). Three patients (12%) showed radiological responses and 22 patients (88%) showed stable disease. One complete response was observed within the eight patients with myxopapillary grade I ependymoma. The remaining responses (six partial responses and one complete response) were observed within patients with more aggressive spine tumors. Of note, the majority of patients with spine tumors experienced a clinical benefit consisting of pain reduction (62%), loss of control of bladder/bowel (73%), and numbness/tingling reduction (57%).

In the overall population, neither tumor grade, nor tumor localization, nor prior systemic treatment received significantly affected the PFS on univariate analyses (83).

Regarding treatment-related toxicity, there was a significant rate of myelotoxicity represented mainly by neutropenia, leukopenia, and thrombocytopenia (7 cases of grade 3/4 thrombocytopenia and 18 cases of grade 3/4 leukopenia/neutropenia) (83).

To date, this study represents the larger prospective series assessing a systemic treatment in patients with spinal cord ependymoma. The foreseeable limits of the study are related to the small number of patients with spinal cord ependymomas and the heterogeneity of this cohort (previously treated/untreated patients and different tumor-grade tumors). Toxicity is another issue that may reduce the use of this combination in clinical practice, even if myelotoxicity was significantly reduced by escalating TMZ dosing after two cycles of treatments.

Another phase II study carried out by Chamberlain MC investigated the role of oral etoposide in 10 patients with recurrent spinal ependymoma. In this study, mPFS and mOS were 15 months (range 2.5–45) and 17.5 months (range 3–45), respectively (84).

Except for these phase II studies, the majority of data investigating systemic treatments came from small retrospective series and single case reports.

Retrospective case series suggested a potential role of TMZ and or other alkylating agents, including cisplatin and cyclophosphamide (86, 88).

A single retrospective study also suggested a clinical benefit with bevacizumab (90).

To date, basket trials are now recruiting adult patients with spinal cord ependymoma and testing brigatinib (a target agent active on tumors cells with EML4-ALK translocation) or neratinib (an EGFR and HER 2 inhibitor) in patients with neurofibromatosis type 2-associated progressive tumors (NCT04374305).

Similarly, selumetinib (a MEK inhibitor) is being tested in a similar population (NCT03095248).

In the immunotherapy era, preclinical studies identified ependymomas as tumors with an immune-suppressive phenotype mainly composed of exhausted T cells (91, 92). The only case report investigating the programmed death 1 (PD1) inhibitor tislelizumab in a metastatic myxo-papillary ependymoma resulted in a durable stable disease with a PFS of 18 months (87).

To date, a phase II study is currently investigating the PD1 inhibitor nivolumab (NCT03173950) in adult patients with rare central nervous system tumors including spinal ependymoma.

Other immunological treatments consist of CAR-T therapy. These engineered T cells have achieved important outcomes in solid and hematologic malignancies and represent a concrete hope also in patients with central nervous system tumors (93–95). The majority of trials investigating CAR T are tailored to pediatric patients. The NCT04661384 is a phase I trial investigating the IL13Ralpha2-CAR T cells within patients with leptomeningeal involvement from ependymoma, glioblastoma, and other malignancies. The accrual is opened also for adult patients.

## 9 Conclusions

Adult spinal ependymomas are rare tumors. However, important molecular insights have been gained in recent years. Optimal treatment requires surgery with the goal of gross total resection and radiotherapy when indicated. No standard systemic treatment has been established, although promising results have been achieved with the combination of TMZ + lapatinib. Promising and new treatments are being investigated, such as brigatinib, selumetinib, and neratinib together with immunotherapy strategies, including CAR-Ts.

Dedicated neurological supportive care and a multidisciplinary approach must always be favored to achieve optimal disease management, limit toxicity, and preserve quality of life.

## Author contributions

GC: Writing – original draft, Writing – review & editing. FP: Writing – original draft. EF: Writing – original draft. VB: Writing – original draft. AS: Writing – original draft. MP: Writing – original draft. RM: Writing – original draft. VD: Writing – review & editing. BB: Writing – review & editing. GLi: Writing – original draft. MC: Writing – original draft. MS: Writing – review & editing. MM: Writing – review & editing. GM: Writing – original draft. PN: Writing – original draft. GLo: Writing – original draft, Writing – review & editing.
